# Epidemiology of snakebites in Kédougou region (eastern Senegal): comparison of various methods for assessment of incidence and mortality

**DOI:** 10.1186/s40409-016-0064-9

**Published:** 2016-03-16

**Authors:** Absa Lam, Bouna Camara, Oumar Kane, Amadou Diouf, Jean-Philippe Chippaux

**Affiliations:** Centre Antipoison, Ministère de la Santé et de l’Action Sociale, Dakar, Sénégal; Service de Réanimation Centre Hospitalier National de FANN, Dakar, Sénégal; Laboratoire de Toxicologie et d’Hydrologie, Faculté de Médecine, de Pharmacie et d’Odontologie/UCAD Dakar, Dakar, Sénégal; UMR 216, Mother and Child Facing Tropical Diseases, Institut de Recherche pour le Développement, Cotonou, Bénin; Université Paris Descartes, Sorbonne Paris Cité, Faculté de Pharmacie, Paris, France

**Keywords:** Epidemiology, Snakebite, Envenomation, Senegal, Incidence

## Abstract

**Background:**

Although considered a public health issue in Senegal, the actual incidence and mortality from snakebite are not known. In the present study, an epidemiological survey was carried out in Kédougou region, southeastern Senegal, where envenomations, particularly by *Echisocellatus*, are frequent and severe.

**Methods:**

Three sources of data were used: records from health centers and reports by health professionals; traditional healers; and household surveys.

**Results:**

The annual incidence and mortality provided by health centers were 24.4 envenomations and 0.24 deaths per 100,000 population, respectively. The annual incidence recorded by traditional healers was 250 bites per 100,000 inhabitants, but the number of deaths was unknown. Finally, the household surveys reported an annual incidence of 92.8 bites per 100,000 inhabitants and an annual mortality rate of 2.2 deaths per 100,000 inhabitants. The differences in incidence and mortality between the different methods were explained by significant bias, resulting in particular from the complex patient’s healthcare-seeking behavior. The incidence provided by health records should be used to specify the immediate quantitative requirements of antivenoms and places where they should be available first.

**Conclusion:**

Mandatory reporting of cases would improve the management of envenomation by simplifying epidemiological surveys. Patients’ preference for traditional medicine should prompt health authorities to urge traditional healers to refer patients to health centers according to defined clinical criteria (mainly edema and bleeding or neurotoxic symptoms). Finally, household surveys were likely to reflect the actual epidemiological situation. Poison Control Center of Senegal should continue its work to sensitize stakeholders and train health staff.

## Background

Although widely regarded as a public health issue, there is no consolidated data on the number of victims or severity of snakebite envenomation in Senegal. Some specific studies evaluated the annual incidence up to several thousand cases and a few hundred deaths, without further precision [1–4]. They all emphasized the lack of accessibility of antivenoms and, in particular, poor supply and positioning of stocks – often found in large cities whilst the need of antivenoms is predominant in rural areas [1].

The assessment of the incidence and mortality due to envenomation is essential to improve the policy of envenomation control and enable the management of preventive and therapeutic measures, starting with the provision of appropriate antivenoms in adequate quantities accessible wherever they are needed for case management [5]. However, in the absence of mandatory reporting of cases, it is necessary to use indirect methods which, although providing consistent estimates, remain approximate [6, 7]. We can schematically oppose two sources of data: health statistics and household surveys. The first, whether retrospective or prospective, is limited to patients seen in health centers, representing between 40 and 60 % of snakebite victims, whose records are often incomplete [8]. The second, probably more accurate and reliable, requires a rigorous methodology [6]. In addition, it raises the problem of the representativeness of surveyed localities [7].

The use of information provided by traditional healers has been proposed by some authors [9–11]. Traditional healers treat most of victims, if not all, and may constitute a remarkable data source. However, very few of them keep reliable registers, and the records are most often based on their memory, which can cause significant bias.

The objective of this study was to describe and compare, in a regional survey, epidemiological, clinical, and therapeutic information from three different sources: health centers and their staff, traditional healers and household surveys.

## Methods

**Fig. 1:**
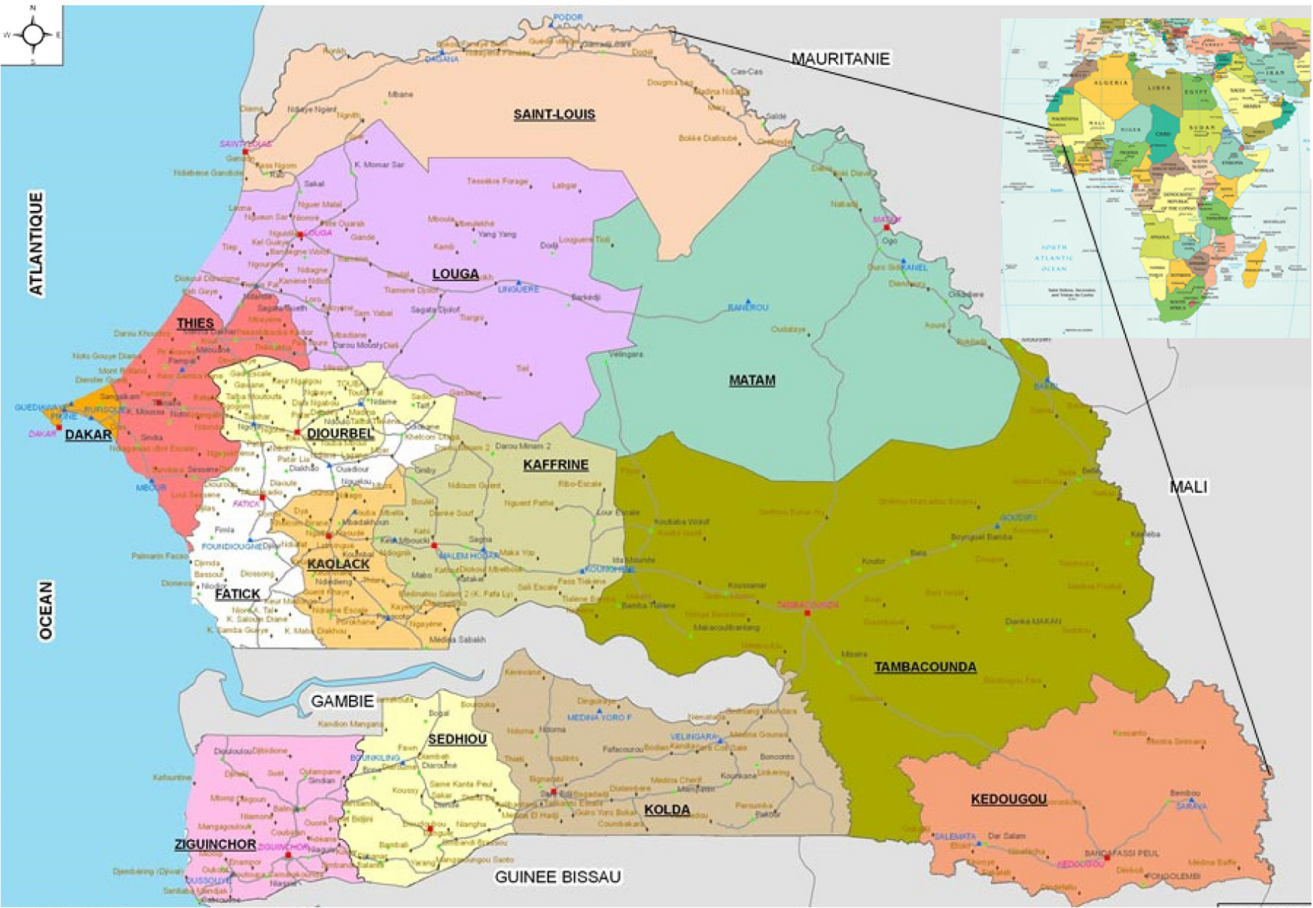
Map of Senegal showing the survey location

The new Kedougou region, south of the Tambacounda region, covers 16,816 km^2^. Its population was estimated in 2008 at 120,000 inhabitants with a density of seven inhabitants per km^2^. This area is limited to the east by Mali, west and north by the region of Tambacounda and south by the Republic of Guinea (Fig. 1). The region comprises three departments: Kédougou, Saraya and Salémata. Its geographical location, its relief, its tropical climate and its wetland ecosystem, explain the wide diversity of snake species, notably with an abundance of *Echisocellatus*, a Viperidae whose venom is inflammatory, hemorrhagic and necrotizing [12].

The retrospective study of snakebites covered the years 2004 to 2009. Surveys involved three target groups:

- Health facilities, i.e., records from health centers and reports from health workers, to whom a questionnaire was administered to assess the management of cases of snakebites recorded during the study period. In the records, the date of consultation, gender, age, origin, site of the bite, treatment, evolution and possibly other available observations were noted. This also allowed to obtain information about the availability of medical resources, particularly antivenoms.
- Traditional practitioners were identified by local authorities and people interviewed during household survey; a questionnaire was administered on their professional practices.
- Households were randomly drawn according to the WHO model, which requires sampling from 30 clusters of ten households, each from an exhaustive list of localities. All inhabitants of these households were included in the survey. Victims and relatives of deceased victims present at the time of the survey were asked for the circumstances and consequences of the bite. In addition, local authorities of the 57 main localities of the region and those in sampled households were interviewed.

In order to sensitize the local staff involved in the support and logistics, workshops were organized to train investigators on the methodology of fieldwork. Standardized questionnaires were used for the surveys in health structures, traditional healers and households.

Data analysis was carry out using Epi info® software.

## Results

**Fig. 2:**
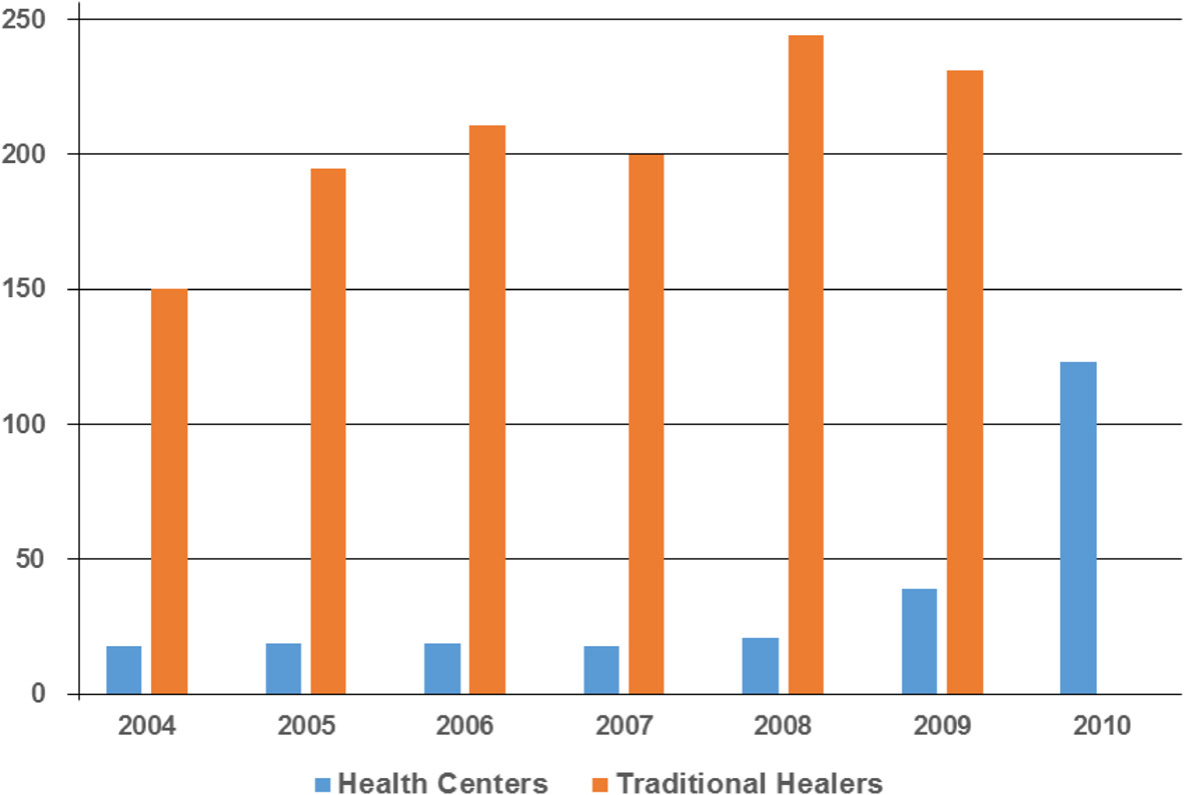
Number of snakebites managed by the modern health practitioners and traditional healers

Medical records were investigated and 31 health workers were surveyed. A total of 122 cases were treated (20.3 cases per year on average, i.e., an annual incidence of 24.4 per 100,000 inhabitants). The evolution of the number of snakebites increased from 2007 (Fig. 2). Adults accounted for 62 % of victims and nearly 65 % were male. The site of the bites was not specified in 86 % of cases. More than half of the patients received only outpatient treatment and one in ten patients was hospitalized or referred. Antivenom was used in 19 % of cases; however, no further details about the type or trade name was recorded. The outcome was unknown in 71 % of the patients, good in 26 % of cases, and sequelae were observed in 2 %. Case fatality rate was close to 1 % (mortality = 0.24 deaths per 100,000 inhabitants).

Sixty interviewed traditional healers treated 1250 snakebites (annual incidence = 250 per 100,000 population). The annual distribution was roughly constant between 2004 and 2009 (Fig. 2). Actually, traditional healers unexperienced in snakebite treatment transferred the patients to peers, and only one quarter of patients to health centers. However, none of traditional healers held patient records to follow up the patients. Moreover, none specified the severity of envenomation or indicated the number of deaths.

Household survey reported 464 cases (annual incidence = 92.8 per 100,000 inhabitants) of which only 121 were found. The majority of the victims were adult males, mainly farmers. The circumstances of the accident were related to field work in 66 % of cases. The survey showed that 76.8 % consulted a traditional healer, most of them (70 %) in first intention. The outcome was unknown in 52.4 %, good in 17.2 % of cases and death in 2.4 % (annual mortality = 2.2 per 100,000 inhabitants).

## Discussion

The wide disparity in incidence and mortality between the different sources of information was anticipated. It confirmed the results obtained by other studies and was explained by several factors [8]. It was likely to result from the healthcare-seeking behavior of the patients.

The low incidence and mortality recorded by health statistics were related, mainly, to the low attendance at health centers, leading to a likely underestimate of the incidence. Most cases, if not all in hospitals, were envenomations, which excluded asymptomatic or mild cases representing up to 50 % of patients [8]. The increasing number of cases treated in health centers – and reduction of those managed by traditional practitioners (Fig. 2) – from 2009 on reflected a greater sensitization of health personnel through the action of the Poison Control Center of Senegal that organized training in the whole Senegal and better reporting of cases. However, this does not seem to generate a decrease in the number of victims that seek traditional healers.

The consultation records showed many gaps, particularly with regard to the clinical description, treatment and evolution of envenomation, which contributed to provide limited confidence in these results. Nevertheless, the number of cases received in health facilities expressed the immediate and unavoidable need of antivenoms that should be considered by health authorities for the antivenom supply in order to ensure appropriate management of snakebites.

**Fig. 3:**
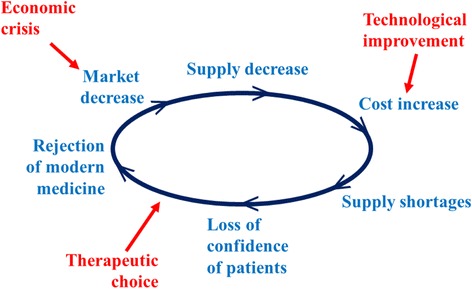
Vicious circle of distribution of antivenoms showing the reduction of accessibility (modified from [13])

The majority of snakebite victims consulted a traditional healer first. These healers are considered more accessible than health workers, cheaper than modern medical treatment, especially if antivenom was administered, and reassured patients who considered snakebites supernatural events requiring appropriate mystical management. According to Chippaux [13], a vicious circle strengthens the role of traditional healers and leads to progressive deny of modern medicine, including antivenom immunotherapy, both inaccessible and poorly used by health workers (Fig. 3). The high incidence of snakebites observed among traditional healers could have several causes. First, they received nearly all patients, including asymptomatic and mild envenomations. Secondly, the faulty memory can lead to some duplicates (the same patient counted several times as a result of numerous consultations in case of sequelae, for example). Third they could take into account patients treated outside the period covered by the investigation. In addition, patients could come from places outside the surveyed administrative region, based on the reputation of the traditional healer, which artificially increased the numerator. The exaggeration of the number of cases is likely. The very favorable trend of clinical evolution and the lack of declaration of deaths can be explained, firstly, by deaths occurring outside the facilities of traditional healers and on the other hand, by a denial from the traditional healer who attributes any adverse evolution, particularly death, to a separate cause of the envenomation, such as a spell or patient misbehavior which could “absolve” the traditional healer from his own liability.

The incidence and mortality obtained from household surveys appeared to be a reasonable compromise between the two other sources of information. However, it is interresting to note that about 20 % of patients did not consult traditional healers, suggesting that patients had self-medicated or, at least part of them, went directly to modern health centers. However, the incidence is four times higher than that displayed by the health statistics, which is consistent with the healthcare-seeking behavior of snakebite victims and their preference for traditional medicine. The incidence was likely to be conservative because some victims of snakebites, with mild clinical symptoms or even asymptomatic, might have forgotten to report the bite. In contrast, mortality ten times higher than that reported in health statistics is consistent with the lack of information on the evolution of envenomation in health centers (71 %) and the preference of some families to bring home patients whose condition is worsening. This did not exclude a bias due to a memory lapse that would lead to mention the patients who died outside the period covered by the investigation. A snakebite is sufficiently traumatic to leave a vivid memory in a person, even if the date and the precise circumstances may fade with time. In this respect, one might prefer interviewing people about recent events (e.g., occurred only during the last year), which promotes good restitution of memory but reduces the total number of observed events. Conversely, to question people about events occurring during their whole life (and establish a yearly average by dividing the number of events by the age of the interviewee) avoids the error related to accidents occurring outside the period study, but exposes to reduce the reliability of details, including the circumstances of the bite, symptomatology and evolution of envenomation. However, the methodology of household surveys should be rigorous. It is necessary, in particular, that the survey sites are randomly selected (or exhaustive) to avoid conducting surveys in localities with very high incidence and therefore not representative of the actual epidemiological situation.

The epidemiological profile of snakebites recorded in health facilities was similar to that described in the household surveys. In addition, it reflected the observations made in most studies in sub-Saharan savannah region [8]. Male adults are mostly affected. Snakebites occurred mainly in rural areas where therapeutic means are limited. However, the estimated case-fatality rate in health centers (about 1 %) may underestimate the actual mortality from snakebites, especially as the use of antivenom was infrequent and the final outcome of the patients was unknown in 71 % of cases. Indeed, it is known that in Africa families remove patients from the hospital and bring them back home if the clinical condition worst and seems to progress to the death for cultural and financial reasons. This is supported by the higher mortality rate of data collected in the household surveys (2.4 % instead of 1 %). As a consequence, it should be preferable to present case-fatality rate results with caution involving this possibility.

The antivenom treatment was used in 19 % of cases due to the poor accessibility of the product. The cost of antivenom and ignorance of its use by health workers led to a shortage of supply that explain the vicious circle described by Chippaux [13]. Reversing this vicious circle by means of financial support of antivenom distribution and training of health workers is undoubtedly a necessary condition for improving the management of snakebites in Senegal.

## Conclusion

The objective of this work was not to specify the incidence and mortality of envenomation by snakebites, but validate epidemiological evaluation methods. The comparison of the different assessment methods of the incidence and severity of envenomation showed the characteristics and strengths of each approach. If data from health statistics proved incomplete, this did not reduce their interest, including specifying the immediate medical needs, namely both quantitative and qualitative, including geographical. Mandatory reporting of cases could simplify the process of epidemiological data acquisition, and thus should be recommended.

Traditional healers, although in the core of the healthcare system and very first recourse of most snakebite victims, produced highly biased and exaggerated information. However, they should be urged to provide first aid and refer patients – on criteria to be clarified – to a modern health center.

The household survey appears as the most reliable and most reasonable source of data, provided that the methodology is respected. The household surveys, for their simplicity and low cost, are an interesting alternative to the pending mandatory case reporting, leading to a better management of envenomation by health structures. They should be preferred and extended to other rural areas of Senegal.

The action of the Poison Control Center of Senegal remains an important asset in sensitization, training and management of snakebites. Its role in access to epidemiological data, improving the accessibility of antivenoms and training of health personnel is critical.

### Ethics approval

This study was a retrospective study based on records from health centers or population surveys that did not involve intervention in patients or human subjects. In addition, it was a study commissioned by the provincial health authorities and authorized by the Ministry of Health of Senegal.
